# Life-Threatening Noninfectious Complications of Peritoneal Dialysis in an Infant with End-Stage Kidney Disease

**DOI:** 10.3390/pediatric17050100

**Published:** 2025-10-01

**Authors:** Chao-Ting Teng, Yi-Hsuan Tang, Hsin-Hui Wang, Yu-Sheng Lee, Chin-Su Liu, Pei-Chen Tsao, Meei-Chyi Guo, Hui-Lan Chen, Chien-Hung Lin

**Affiliations:** 1 Division of Pediatric Immunology and Nephrology, Department of Pediatrics, Taipei Veterans General Hospital, Taipei 11217, Taiwan; 2 Institute of Emergency and Critical Care Medicine, School of Medicine, National Yang Ming Chiao Tung University, Taipei 11217, Taiwan; 3 Department of Pediatrics, Faculty of Medicine, School of Medicine, National Yang Ming Chiao Tung University, Taipei 11217, Taiwan; 4 Division of Neonatology, Department of Pediatrics, Taipei Veterans General Hospital, Taipei 11217, Taiwan; 5 Division of Pediatric Surgery, Department of Surgery, Taipei Veterans General Hospital, Taipei 11217, Taiwan; 6 Division of Transplant Surgery, Department of Surgery, Taipei Veterans General Hospital, Taipei 11217, Taiwan; 7 Institute of Physiology, School of Medicine, National Yang Ming Chiao Tung University, Taipei 11217, Taiwan; 8 Department of Nursing, Taipei Veterans General Hospital, Taipei 11217, Taiwan; 9 Department of Pediatrics, Keelung Hospital, Ministry of Health and Welfare, Keelung 20120, Taiwan

**Keywords:** infant, life-threatening, noninfectious complications, peritoneal dialysis

## Abstract

**Background**: Noninfectious complications of peritoneal dialysis (PD) are common in infants. Mechanical dysfunctions with abdominal compartment syndrome, hydrothorax with respiratory failure, and medication-induced chyloperitoneum are rare during PD. In this case report, we aim to present several life-threatening events and the timely management of a PD infant. **Case Presentation**: This male infant is a case of infantile nephronophthisis, NPHP3/renal-hepatic–pancreatic dysplasia type 1, with end-stage kidney disease, and he received PD therapy at 4 months of age. Because of the young age with low body weight and hepatosplenomegaly with a limited abdominal cavity, intra-abdominal pressure-associated noninfectious complications frequently occurred. Acute respiratory failure with abdominal dullness was detected at 5 months of age. Abdominal compartment syndrome caused by PD catheter outflow obstruction from omental wrapping was diagnosed via laparoscopic revision surgery. Hyperkalemia, decreased PD drainage volume, and sudden respiratory distress occurred at 10 months old. Hydrothorax due to pleuroperitoneal communication was confirmed by scintigraphy. After thoracoscopic diaphragmatic bleb repair and plication surgery were performed, no recurrence of hydrothorax was observed. Calcium channel blocker-induced chyloperitoneum was observed at 13 months of age. Chylous ascites disappeared after tapering off the calcium channel blocker in 3 days. After the patient grew up with a larger peritoneal cavity, no more pressure-associated complications of PD occurred. **Conclusions**: The key to successful treatment of rare and life-threatening noninfectious complications of PD in young infants lies in early detection and timely intervention. A limited abdominal cavity is not a contraindication for PD therapy, especially in very young infants with low body weight, because hemodialysis is not a choice of long-term dialysis modality.

## 1. Introduction

Peritoneal dialysis (PD) is one of the main choices of dialysis modality in pediatric patients, especially neonates and infants, with acute kidney injury and chronic kidney disease. 92.5% of patients less than 1 year old and 51.5% of patients aged 1 to 5 years old with end-stage kidney disease (ESKD) used PD as the initial modality of renal replacement therapy [1]. Advantages of PD in infants and neonates over hemodialysis are its safety with stable intradialytic hemodynamics, good residual renal function preservation, less inflammatory effects with biocompatible peritoneal membrane as the dialyzer, easy device implementation without vascular access, simple technical requirement with less equipment support, a less restricted diet, and cost effectiveness [2,3]. The limitations of PD include meeting the peritoneal membrane and cavity requirements, and slower ultrafiltration and smaller solute clearance than hemodialysis or continuous renal replacement therapy.

Infectious complications of PD contribute to early technique failure, morbidity, and mortality in ESKD pediatric patients. The incidence rate of peritonitis and PD catheter-related infection is decreasing from 0.79 episodes per patient during the years 1992–1996 to 0.44 during 2007–2018, due to improving PD care techniques, patients’ education and training, and antibiotic therapy [4]. On the other hand, the rate of noninfectious complications varied. The incidence rate of noninfectious complications of PD reported in children was 30–57.8%, and of PD tube revisions due to mechanical dysfunction was 17–60% [5,6]. There are many types of noninfectious complications, including PD catheter-related mechanical dysfunction, increased intra-abdominal pressure-related leakage or pain, and dialysate-related metabolic effects [7]. The severity of noninfectious complications varies depending on whether it is caused by hemodynamic or metabolic effects. Early detection and early intervention are important for clinical physicians to avoid and prevent PD failure, morbidity, and even mortality.

We aim to present the case of an ESKD infant with several life-threatening noninfectious complications of PD and the management strategies in this case report.

## 2. Case Presentation

**Figure 1:**
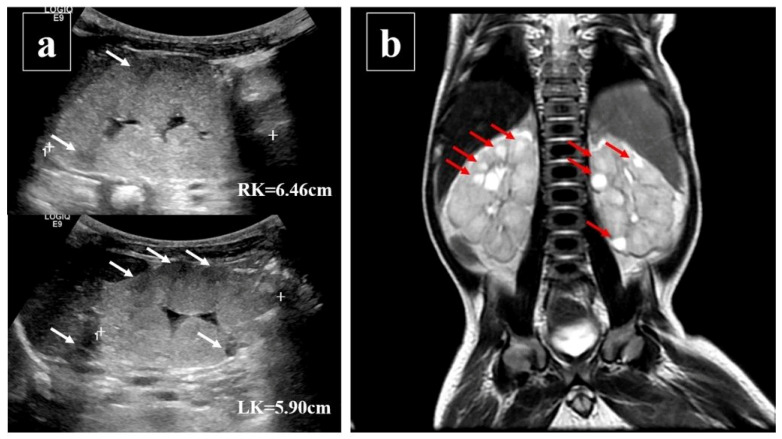
Radiological images of multicystic kidneys. (**a**) Renal ultrasound of multiple renal cysts (white arrows) and hyper-echoic renal parenchyma with loss of corticomedullary differentiation. (**b**) Renal MRI compatible with multicystic kidney disease according to the T2-weighted images (red arrows).

This male term infant presented suffered from poor body weight gain, shortness of breath, and severe metabolic acidosis when he was 27 days old. The diagnosis of multicystic kidneys was confirmed by renal ultrasound and magnetic resonance imaging (Figure 1a,b), and infantile nephronophthisis, NPHP3/renal-hepatic–pancreatic dysplasia type 1, was diagnosed by whole exome sequencing. The whole exome sequencing showed compound heterozygous, truncating mutations in the *NPHP3* gene: c.1817G>A, p.Trp606Ter, heterozygous, likely pathogenic (father’s gene), and c.3402_c.3403delTG, p.Ala1135SerfsTer5, heterozygous, likely pathogenic (mother’s gene).

Due to the progression of ESKD, he started to receive continuous ambulatory peritoneal dialysis (CAPD) treatment at 4 months old, weighing 6 kg. Currently, he is 1 year and 6 months old and weighs 11 kg. He is still undergoing CAPD treatment, which has been successful. Due to the very young age, low body weight, and genetic disease-related abdominal condition with hepatosplenomegaly, several non-infectious complications of PD were observed during the treatment course. Here, we present the rare life-threatening non-infectious complications of PD in an infant with infantile nephronophthisis. Informed written consent was given by his parents for the following clinical data and radiologic images.

**Figure 2:**
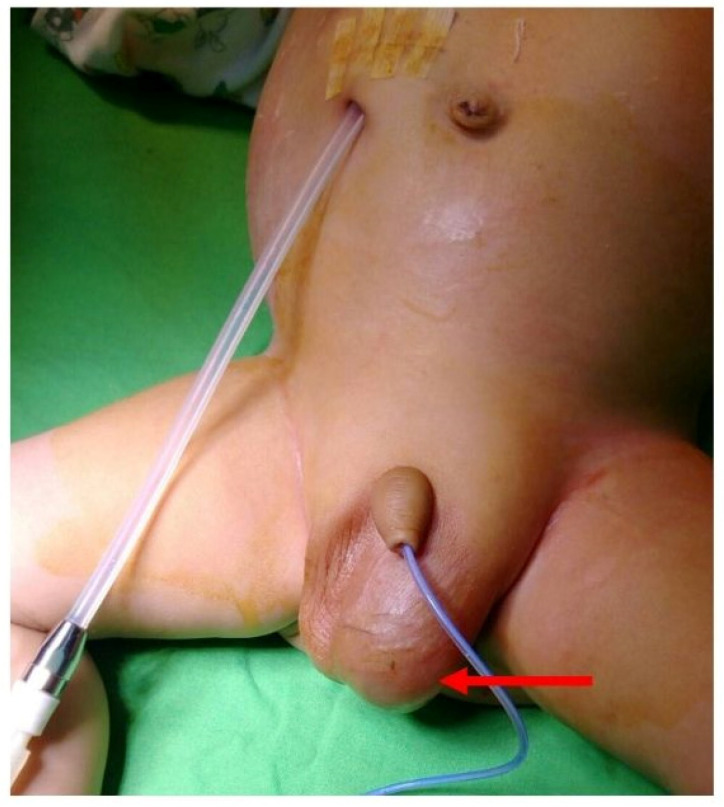
Unilateral hernia with hydrocele on the left side scrotum (red arrow).

**Figure 3:**
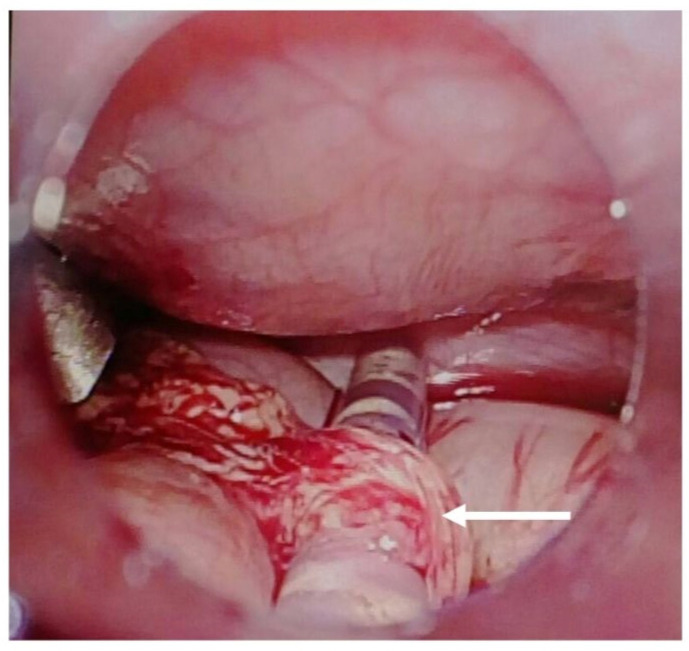
Laparoscopic surgery of omental wrapping (white arrow) over original PD tube.

This patient started CAPD treatment when he was 4 months old. The dialysis prescription, including the volume and dextrose concentration of PD solution, has been adjusted gradually based on both clinical abdominal conditions and the measure of ultrafiltration and dialysis adequacy. When he was 5 months old, physical examination revealed abdominal dullness, an enlarged left scrotum (Figure 2), and a positive transillumination test. A unilateral hernia with hydrocele on the left side of the scrotum was diagnosed. In addition, a sudden decrease in PD ultrafiltration volume with progressing abdominal dullness was also noticed 3 days after the diagnosis of left inguinal herniation. Hyperkalemia, hyponatremia, lethargy, compensated tachycardia, and tachypnea were also noticed. The abdomen X-ray showed the PD catheter was at its correct position without pneumoperitoneum. Under the assumption of omental wrapping of the PD catheter with abdominal compartment syndrome and an inguinal hernia suspected, surgical intervention was performed. PD tube revision for omental wrapping, original PD tube (Figure 3), intra-abdominal pressure reduction, and high ligation for unilateral inguinal hernia, indirect type with hydrocele, were performed successfully. After surgery, his PD adequacy and ultrafiltration returned to a satisfactory state, and he was discharged at 6 months old.

**Figure 4:**
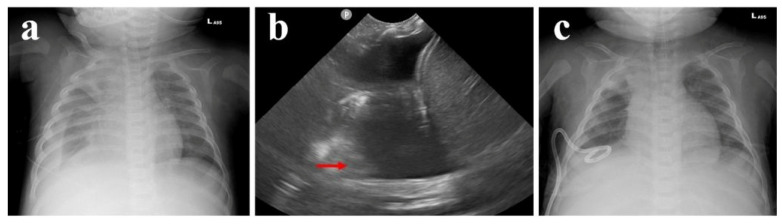
Radiography and ultrasound of pleural effusion. (**a**). Chest x-ray and (**b**). pleural ultrasound with massive right pleural effusion and collapsed right lower lung (red arrow). (**c**). Chest X-ray after pigtail tube insertion.

**Figure 5:**
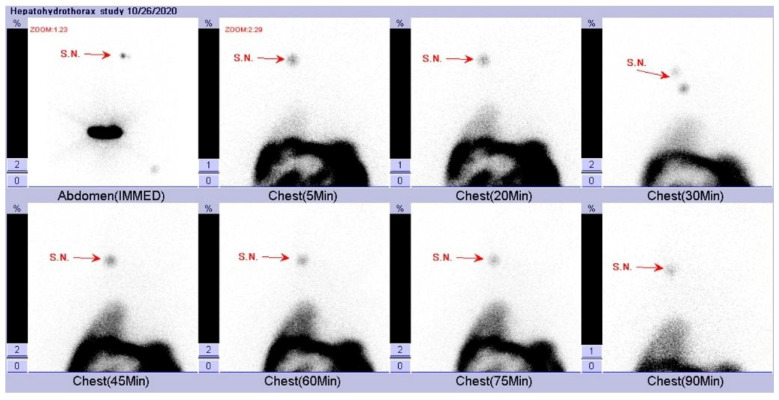
Scintigraphy of abdomen and chest showed radiotracer (Tc-99m-phytate) distribution from peritoneal cavity into right pleural cavity with spared left pleural cavity. IMMED: immediate, Min: minutes, S.N.: standard normalization.

**Figure 6:**
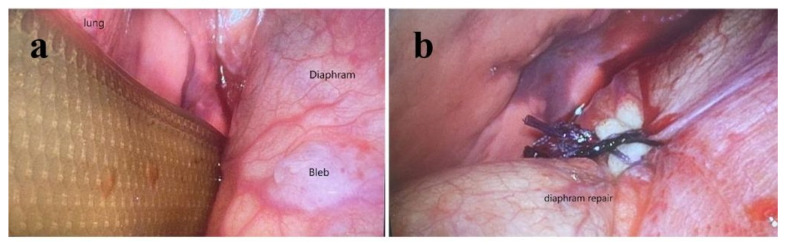
Thoracoscopic surgery for pleuroperitoneal communication. (**a**) Right hemidiaphragmatic bleb. (**b**) Diaphragmatic bleb repair with 3-0 Vicryl suture.

When he was 10 months old, he was admitted to the pediatric intensive care unit for CAPD-related peritonitis with septic shock. After optimal intravenous and intraperitoneal antibiotics treatment, his clinical condition improved and became stable in **a** month. However, acute dyspnea with compensated respiratory distress was noted. Persistent hyperkalemia and decreasing PD drainage volume were also noted. Chest X-ray showed right pleural effusion with a collapsed right lung and blunting of the right costophrenic angle (Figure 4a). On top of that, pleural ultrasound also revealed right-sided pleural effusion and collapsed right lower lung (Figure 4b). After the insertion of a pigtail tube into the right pleural cavity (Figure 4c) with drainage of 90 cm^3^ clear pleural effusion, his respiratory distress was relieved. Because of the acute occurrence of unilateral pleural effusion, pleuroperitoneal communication was suspected. We compared the laboratory biochemistry data of pleural effusion, dialysate fluid, and serum (Table 1), which revealed a transudative effusion and no high glucose gradient between pleural effusion and serum. Pleural effusion and dialysate fluid cultures showed no bacterial growth. We also arranged scintigraphy, which showed evidence of leakage of fluid in the abdominal cavity into the right pleural cavity (Figure 5). Surgical intervention with thoracoscopically assisted diaphragmatic repair and plication was performed successfully. A 0.5 × 0.5 cm bleb on the anterolateral right hemidiaphragm surface was noted with progressive leakage of peritoneal dialytic fluid during operation (Figure 6a,b). After post-operative care, he was discharged.

**Figure 7:**
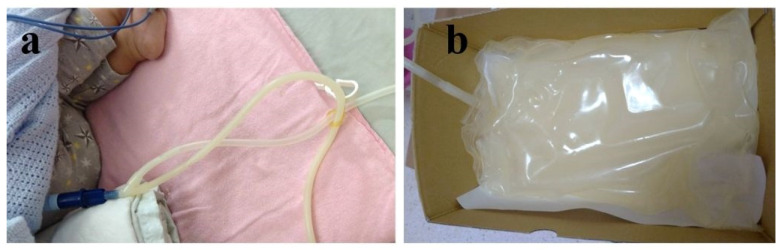
Chylous ascites in dialysate fluid drainage tube set (**a**) and bag (**b**).

He was admitted again at 1 year and 1 month old with 9 kg due to poor blood pressure control, decreasing PD drainage volume, and poor appetite with vomiting. Amlodipine, one of the dihydropyridine calcium channel blockers (CCB), was titrated up to 3.75 mg three times per day for hypertension control. An abdominal X-ray was performed and showed no displacement of the PD tube. The PD drainage volume increased after position adjustment by altering his body and trunk position to facilitate PD outflow. One week later, milky-color dialysate fluid was noted (Figure 7a,b). Laboratory biochemistry data of dialysate fluid and serum (Table 2) revealed true chylous ascites. Infection, malignancy, trauma, pancreatitis, or tuberculosis were not likely causes. We traced back his medication. Amlodipine was tapered back to 2.5 mg three times per day, and doxazosin was added for hypertension control. Chyloperitoneum was relieved 3 days later. He was then discharged under stable conditions. After that, he demonstrated an adequate dialysis dose, steady body weight gain from 9 kg at 1 year of age to 12 kg at 3 years, and progressive improvement in developmental milestones during regular follow-up in the pediatric nephrology outpatient clinic.

## 3. Discussion

Noninfectious complications of PD are common in infants with ESKD and may result in modality failure [8]. They are usually classified as catheter-related, increased intra-abdominal pressure-related, metabolic-related, and others [6,7,9,10]. Although they do not seem to be as severe as infectious complications, noninfectious complications, such as cardiopulmonary function involvement or severe electrolyte imbalance, may also be life-threatening.

PD catheter-related noninfectious complications include bleeding, dialysate leakage, tip migration, tube obstruction/kinking, side/tip holes adhesion, and organ injury/perforation [11,12,13]. LaPlant MB et al. reported catheter-related noninfectious complications as follows: leakage (21%), adhesions (6%), and migration (4%) in infants [12]. Imani PD et al. reported leakage (32%), blockage/obstruction (26%), and malposition (18%) in young children [11]. Borzych-Duzalka D et al. reported that the causes of PD catheter revision in children were mechanical malfunction (60%), peritonitis (16%), exit site infection (12%), and leakage (6%) [5]. Most of the catheter-related complications do not necessarily require emergent surgical intervention. However, the diagnosis of peritoneal dialysis catheter outflow (so-called one-way) obstruction may be delayed because of intact dialysate inflow. Omental wrapping is one of the causes of early outflow obstruction after PD catheter implantation. The incidence rate of omental wrapping-induced malfunction was around 4.5–15% [14]. Therefore, prophylactic omentectomy during PD tube implantation was reported [12]. Progressing outflow obstruction can cause abdominal compartment syndrome with multiorgan failure, resulting in high morbidity and mortality. The risk factors for abdominal compartment syndrome are age under 1 year, previous cardiac surgery, extracorporeal support, septic shock, burn, and intra-abdominal infection or hemorrhage [15,16]. In this critically ill infant patient, continuous infusion of peritoneal dialysate into the abdominal cavity is required to maintain cardiorespiratory function and preserve fluid, electrolyte, and acid–base balance, so close monitoring of intra-abdominal pressure via bladder pressure assessment was unreliable, as the continuous infusion of peritoneal dialysate into the abdominal cavity interfered with measurement accuracy [16]. Therefore, the cardiorespiratory status change, increased abdominal circumference, and poor distal extremities perfusion are vital to early detection by clinical physicians. Intra-abdominal pressure decompression is the goal of treatment, and percutaneous drainage and decompressive laparotomy/laparoscopy surgery can be arranged based on patients’ clinical conditions [15,16,17]. In the case of our patient, he had early abdominal compartment syndrome 1 month after PD catheter implantation with compensated cardiopulmonary function, and PD malfunction with electrolyte imbalance. Without timely management, life-threatening multiorgan failure due to PD tube outflow obstruction-induced abdominal compartment syndrome and PD failure may occur. Therefore, prompt surgical intervention with catheter revision is important for resolving omental wrapping outflow PD catheter obstruction.

Peritoneal dialysis itself may result in increasing intra-abdominal pressure due to increased volume of PD solution and hypertonic dialysate containing high dextrose concentration [18]. Aksoy GK et al. reported 10.6% abdominal distension, 23.2% hernia, and 0.6% hydrothorax in intra-abdominal hypertension-related noninfectious complications [6]. Intraperitoneal hypertension may develop weak points in the peritoneal muscular layers [19] leading to ventral herniation, inguinal herniation, or pleuroperitoneal fistula located at different points. Herniation may be obviously detected by physical examination, and it does not endanger lives. Timing of repair surgery for herniation is elective, depending on each patient’s condition.

Hydrothorax due to pleuroperitoneal communication is rare, with an estimated prevalence of 0.6–2% in PD patients [6,18,20]. The main hypothesized mechanism is that the pressure gradient between the peritoneal cavity and the pleural cavity is increased because of dialysate volume [21]. It is usually unilateral, predominantly on the right side, and 25% of patients are asymptomatic [18,20]. Acute hydrothorax may possibly progress to respiratory decompensation and even lead to lethal obstructive shock due to sudden massive fluid accumulation and right lung collapse-induced cardiac tamponade. Experience in hydrothorax and immediate intervention for relieving obstruction are important to protect cardiopulmonary function. Unilateral pleural effusion is a crucial hint. Biochemistry laboratory data collected from diagnostic or therapeutic thoracocentesis usually reveal transudative effusion and a higher glucose concentration in pleural effusion than serum glucose level. Chow KM et al. reported that pleuroperitoneal communication is not likely with a low glucose gradient between pleural effusion and serum of less than 2.8 mmol/L [21,22]. Hence, PD-associated hydrothorax is usually referred to as sweet hydrothorax due to the use of glucose-rich dialysates [18,23]. However, in some studies and our patient’s case, pleuroperitoneal communication was observed without a high glucose gradient between the pleural effusion and serum [24,25]. Some hypotheses state that the severity of leakage depends on factors such as the size of the diaphragmatic defect, the glucose absorption rate of the pleural surface, or the delay between the specimen of pleural effusion collection and the dialysate exchange [25]. The most useful diagnostic tool is peritoneal scintigraphy with technetium-99m-diethylene-triamine-pentaacetate [18,26]. The choice of hydrothorax treatment depends on clinical conditions; temporarily suspending PD and switching to hemodialysis as a conservative diaphragmatic defect treatment, chemical pleurodesis, surgical repair by thoracotomy, or video-assisted laparoscopic thoracotomy have all been reported [18,22,25]. In our patient’s case, emergent therapeutic thoracocentesis with pigtail tube insertion and drainage was initially performed for acute respiratory failure. A confirmed diagnosis was made by scintigraphy 2 days after his vital signs became stable. Our repair operation was completed on the third day by video-assisted laparoscopic surgery, and no recurrent hydrothorax was detected at follow-up appointments. Therefore, early diagnosis and early intervention are important for preventing mortality and morbidity and avoiding failure of PD therapy.

Chyloperitoneum during PD is an uncommon complication diagnosed by a triglyceride concentration of more than 1.24 mmol/L in peritoneal fluid [6]. The etiologies of chyloperitoneum can be generally divided into two categories: congenital and acquired [27]. Congenital lymphatic duct anomaly and trauma or surgery-related lymphatic duct damage are the most common causes in pediatric patients [28,29]. CCB-associated chyloperitoneum is rarely reported in PD patients, even though CCB is a common antihypertensive medication used by ESKD patients [30]. Kim S. et al. reviewed that chylous ascites occurred within 4 days of CCB initiation and resolved within 24 h of CCB withdrawal [31]. The hypothesis mechanisms of CCB-induced chylous ascites are lymphatic flow dysfunction in triglyceride disposal and increased ultrafiltration through the peritoneal membrane. The main treatment of CCB-associated chyloperitoneum is to stop or taper CCB [31,32]. However, before stopping or tapering CCB, it is important to exclude other common etiologies, like peritonitis or trauma-related chylous ascites, since the beginning of chylous ascites. Because cloudy, milky, and turbid chylous ascites is usually observed in PD-associated peritonitis, empirical antibiotic therapy is crucial and cannot be delayed. After biochemistry laboratory analysis of ascites and tracing back medication history, CCB-associated chyloperitoneum should be considered. In our patient’s case, peritonitis and trauma history were excluded first, and then we tapered the CCB dosage and prescribed another antihypertensive medication. His chyloperitoneum occurred after 7 days of CCB dose titration and disappeared after 72 h of CCB dosage tapering.

Our patient is a case of genetic disease progressing to ESKD attributed to his young age, low body weight, and hepatosplenomegaly with limited abdominal cavity volume; therefore, the intra-abdominal pressure-associated noninfectious complications frequently occurred. Importantly, as the child grew and achieved increased body weight and expansion of his abdominal cavity, these pressure-associated complications notably decreased. This observation aligns with findings that younger children, particularly infants, are at higher risk of mechanical complications such as catheter leaks, hernias, and flow dysfunction, but these risks decline with age and growth as the abdominal wall and cavity mature [33]. Moreover, in infants treated with PD, catch-up growth occurs in a substantial proportion, and their metabolic and cardiovascular complications, such as left ventricular hypertrophy, may also improve over time [34,35]. The pressure-associated complications decreased as he grew up with increasing body weight and abdominal cavity. Therefore, a limited abdominal cavity is not a contraindication for PD therapy, especially in very young infants with low body weight, for whom hemodialysis is not a choice of long-term dialysis modality. Early initiation of PD can enable stabilization and growth, thereby mitigating many non-infectious complications over time.

## 4. Conclusions

This case report emphasizes the rare but life-threatening noninfectious complications of peritoneal dialysis in infants. The successful treatment for young infants with PD complications depends on early diagnosis and early intervention.

## Figures and Tables

**Table 1:** Hydrothorax: biochemistry data of pleural effusion, dialysate fluid, and serum.

Laboratory Data	Pleural Effusion	Dialysate Fluid	Serum
Protein (g/L)	4	2	47
Glucose (mmol/L)	7.493	25.364	7.604

**Table 2:** Chyloperitoneum: biochemistry data of dialysate fluid and serum.

Laboratory Data	Dialysate Fluid	Serum
Triglyceride (mmol/L)	4.396	3.277
Cholesterol (mmol/L)	0.311	5.569
Albumin (g/L)	2	29
Glucose (mmol/L)	7.493	5.717
Protein (g/L)	6	52
Lactate dehydrogenase (µkat/L)	0.433	3.9
WBC (10^9^/L)	0.002	N/A
RBC (10^12^/L)	0.000027	N/A

WBC: white blood count; RBC: red blood count; N/A: not available.

## Data Availability

The original contributions presented in this study are included in the article. Further inquiries can be directed to the corresponding author(s).

## Abbreviations

CAPD: Continuous ambulatory peritoneal dialysis; CCB: Calcium channel blockers; ESKD: End-stage kidney disease; PD: Peritoneal dialysis
